# Null Effect of Olfactory Training With Patients Suffering From Depressive Disorders—An Exploratory Randomized Controlled Clinical Trial

**DOI:** 10.3389/fpsyt.2020.00593

**Published:** 2020-06-23

**Authors:** Luise D. Pabel, Julia Murr, Kerstin Weidner, Thomas Hummel, Ilona Croy

**Affiliations:** ^1^Department of Psychotherapy and Psychosomatic Medicine, Medizinische Fakultät Carl Gustav Carus, TU Dresden, Dresden, Germany; ^2^Smell & Taste Clinic, Department of Otorhinolaryngology, TU Dresden, Dresden, Germany

**Keywords:** depression, olfaction, olfactory function, threshold, sensory

## Abstract

Due to a close functional relation between brain areas processing emotion and those processing olfaction, major depression is often accompanied by reduced olfactory function. Such hyposmia can be improved by regular olfactory training (OT) over several months. As this training furthermore improves subjective well-being, we explored whether OT is a useful complementary strategy for depression treatment. A total of 102 depressive outpatients were randomly assigned to OT or a control training condition, which were performed twice a day for 16 weeks. Compliance was continuously monitored. Before and after training we measured depression severity and olfactory function. About half of the patients of both groups did not complete the training. Among the remaining patients, depression severity decreased significantly in both groups. The absence of an interaction effect indicated no selective impact of OT and the variance of depression improvement explained by OT was as little as 0.1%. The low compliance suggests that OT is not feasible for large parts of our sample of depressive outpatients, most likely due to a disease-immanent lack of motivation. In those patients who completed the training, lack of specific effects suggest that OT is not more useful then unspecific activation or attention training.

**Clinical Trial Registration:**

This clinical trial was registered at German Registry for Clinical Trials (DRKS), main ID: DRKS00016350, URL: http://www.drks.de/DRKS00016350.

## Introduction

Depressive disorders are one of the most widespread mental disorders worldwide. Nearly 25% of inability to work in Germany is caused by depressive disorders (1). The state and the economy incur an annual cost of up to 21.9 billion euro due to absenteeism, reduced productivity of sick employees and for the treatment of those affected (1). This implies a high demand for treatment options.

Olfactory processing pathways directly feed into structures involved in salience detection and emotion generation and processing, namely the orbitofrontal cortex, the amygdala, and the hippocampus (2). Impaired olfactory function in patients suffering from hyposmia or anosmia relates to reduced central processing of emotion (3). The joint processing pathways may explain the overlap between depressive symptoms and olfactory disorders, which has been shown in animals and humans: in the rodent model, bilateral destruction of the olfactory bulb (OB) reduces serotonin and dopamine concentrations and influences the immune system, resulting in depression-like behavior (4, 5). Antidepressant medication diminishes these behavioral effects (4). Compared to controls, rats from the depression condition exhibit a significantly thinner olfactory epithelium which is characterized by a marked decrease of olfactory receptor neurons (6). The bulbectomized rat is hence used as an animal model for depression (7), and various studies demonstrate that the same mechanisms in principle apply to humans: we found a negative relation between OB volume and depression score in clinical samples (8, 9). Moreover, people with diagnosed depression often exhibit a reduced olfactory capacity: they are less sensitive to odors and have problems in discriminating and identifying them [for an overview see (10, 11)]. This pattern is removable in the course of an antidepressive medication or psychotherapy (12, 13). Most of the research has been done in the field of depression; however some other studies indicate that olfactory abnormalities occur in different mental disorders such as schizophrenia, obsessive-compulsive disorder (OCD), anxiety, or Posttraumatic stress disorder (PTSD) and it has been argued that olfactory function is a marker for mental disorders (14). While in schizophrenia, extensive deficits in odor identification, sensitivity, discrimination, and memory have been reported (15), rather specific domains of olfactory function seem to be affected in other mental disorders. In OCD, the olfactory identification ability seems to be diminished, while olfactory threshold remains unaffected (16), although other research groups found an impairment in all three domains (odor identification, odor threshold, and odor discrimination) in patients diagnosed with OCD (17). Regarding anxiety disorders, there is evidence for significant deficits in olfactory discrimination among patients, while no changes in odor identification and threshold have been found (18, 19). Lastly, studies with war veterans revealed an impairment of olfactory identification in patients suffering from combat-related PTSD (20, 21). At least for the OB volume the effect seems more specific, as only depression and schizophrenia seem to relate to reductions of this structure (22, 23).

Olfactory training (OT)—daily short-term exposure to odors for 12–20 weeks—is an effective method to improve olfactory function. Such training enhances the awareness to odors and fosters the processing of olfaction (24–27). After completion of OT, patients exhibit on average increased OB volume (28, 29), increased responsiveness to odors at the level of the olfactory epithelium (30), and improved olfactory sensitivity (25–27). Such an adaptive enhancement of olfactory sensitivity may indicate an attention-based moderation of olfactory receptor turnover rates and an increase in the number of expressed receptors or the number of olfactory receptor neurons (10). Enhanced olfactory function after successful OT leads to enhanced central olfactory processing and may thereby lower the action potential threshold in subsequent brain structures involved in salience detection and emotion regulation, which makes such structures susceptible for reaction (10).

In patients with impaired cognitive function due to Parkinson’s disease, OT leads not only to enhanced olfactory function, but also to improved verbal fluency (31). Furthermore, the research group of Schöpf and her colleagues showed on the basis of a Functional magnetic resonance imaging (fMRI) study that olfactory function recovers by means of inducing neural reorganization processes with the help of OT in a sample of anosmic patients (32) [for a detailed review on the field of neuroplasticity in olfactory function see (33)].

Short time exposure to odors was also shown to have beneficial effects on mood: In non-clinical subjects, improvements in mood and/or decrease of anxiety have already been reported after exposure to odors [for example (34–36)]. It has been further shown that in patients with major depression, exposure to odors precipitated reductions in required tricyclic antidepressant dose (37). OT has so far not been conducted in patients with depression. A sample of elderly participants aged 50–84 years who completed OT did however not only improve in olfactory sensitivity and discrimination, but also in verbal cognitive function (38). Furthermore, those participants felt younger and more active after training than a control group that solved Sudokus in the same time period (38). Moreover, in a subgroup of participants characterized by subclinical depression at the first appointment, the OT reduced the severity of depression by about four points from a score 14 to a score 10 as measured with the Beck Depression Inventory (38). Those results generate the hypothesis that OT is a useful therapeutic approach for patients with depression. However, OT needs to be conducted twice a day over a long time period, a precondition which may contradict one of the core symptoms of depression, namely lack of motivation. We therefore aimed to investigate in an exploratory trial in a sample of clinically depressed patients a) whether OT is feasible and b) whether it has a positive effect on depressive symptoms. In line with the aforementioned studies (24–27), we furthermore hypothesized that OT improves olfactory function and, as a “side effect”, cognitive function (39).

## Materials and Methods

This randomized controlled clinical trial was registered retrospectively at the German Registry for Clinical Trials (DRKS) on 19/12/2018, the registration number is: DRKS00016350. We apologize for not registering our trial prospectively. Pre-registration is a rather new practice which was not common in olfactory research by the time we started the recruitment of our research project. We hereby confirm that future trials will be registered prospectively.

### Participants

Initially, 102 patients with depressive symptoms, thereof 65 females, were tested (age: *M* = 37.7, *SD* = 12.6, range = 18–78 years). The sample size estimation of 100 participants per group, as determined in the trial protocol in order to statistically ensure an effect of *d* ≥ 0.5 (see **Supplements**), could not be accomplished due to the unpredicted dropout related delay in the recruitment process. A maximum recruitment time frame of 2 years was determined; thus the recruitment process was terminated accordingly. The participants were randomly assigned to an experimental group (OT group; *n* = 53) and a control group (CT group; *n* = 49). A randomization list was thus created by the investigator and the participants were assigned to this list chronologically.

The sample was recruited by the investigator from patients who presented themselves to the psychosomatic outpatient department of the Clinic of Psychotherapy and Psychosomatics, University Hospital Dresden. Specific hypotheses of baseline measurement results (relation between olfactory function, symptom severity, duration, and course of depression) are published elsewhere (40). Symptoms of an at least mild depression [Beck Depression Inventory (BDI)-score >13 and <20] served as inclusion criterion (BDI: *M* = 26.2, *SD* = 9.7, range = 13–59). The variety of depression severity ranging from mild up to severe depression was intended, taking the relative heterogeneity of the sample into account. The included main diagnoses comprised the following mood disorders: depressive episode (F32), recurrent depressive disorder (F33), and adjustment disorder with depressed mood (F43.22) as assigned in the participants´ medical records. The patients were diagnosed by trained psychotherapists during their diagnostic initial interview in the psychosomatic out-patient department and classified with the diagnostic system International Statistical Classification of Diseases and Related Health Problems-10th Revision (ICD-10) (41). The trained clinicians attended regular consensus diagnosis meetings and each diagnosis was confirmed by the head of unit. In the OT group, 25 participants showed comorbid mental disorders, while in the CT group, 24 participants fulfilled the criteria of at least one more mental disorder diagnosis (compare **Table 1**).

**Table 1:** Sample description of the participants that finished the training period of at least 12 weeks, n = 49.

		OT (*n* = 25)		CT (*n* = 24)		p^**^	*d*	
		*M*	*SD*	*M*	*SD*			
Depression severity (BDI)	Pre-test	27.3	10.8	23.5	9.2	.19	.39	
	Post-test	21.1	12.9	17.6	10.1	.29	.31	
Odor threshold	Pre-test	9.7	3.5	10.9	3.4	.25	.36.36	
	Post-test	10.2	3.1	10.5	2.7	.73	.11	
Odor identification	Pre-test	12.8	1.6	12.1	2.3	.23	.36	
	Post-test	12.0	1.9	11.5	2.0	.38	.26	
Importance of olfaction	Pre-test	1.8	0.4	1.7	0.4	.37	.26	
	Post-test	1.8	0.4	1.8	0.5	.70	<.001	
Verbal fluency (RWT-FL)	Pre-test	14.0	3.6	12.4	3.3	.12	.47	
	Post-test	14.2	3.6	12.1	3.1	**.03**	.64	
Attention (d2)	Pre-test	191.0	42.7	191.1	44.6	.99	<.001	
	Post-test	216.0	47.7	212.0	48.2	.77	.09	
Subjective olfactory function	Pre-test	3.6	0.8	4.0	0.7	.10^+^	.54	
	Post-test	3.7	0.6	3.6	1.2	.72^+^	.11	
Age^*^	Pre-test	40.4	12.4	38.0	12.9	.50	.19	
Duration of disease in months^*^	Pre-test	31.1	32.0	44.2	66.2	.38	.26	
Number of diagnoses^*^	Pre-test	1.7	0.95	1.7	0.86	.93^+^	<.001	
		***n***	***%***	***n***	***%***	p^***^		
Sex (female)		17	68.0	13	54.2	.32		
Currently in psychotherapeutic treatment	pPe-test	8	32.0	5	20.8	.38		
	Post-test	13	52.0	11	45.8	.67		
**Diagnosis^+++^**								
Mood disorders (F30-F39)	Depressive episode (F32)	8	33.3	7	28.0	.69		
	Recurrent depressive disorder (F33)	11	45.8	11	44.0	.90		
	Adjustment disorders (F43.2)^++^	5	20.8	7	28.0	.56		
Neurotic, stress-related and somatoform disorders (F40-F49)	Anxiety disorders (F40, F41)	5	20.0	5	20.8	.94		
	Obsessive-compulsive disorder (F42, F60.5)	1	4.2	2	8.0	*Chi²-*test not per-formed due to insuffi-cient sample size *Chi²-*test not per-formed		
	Post-traumatic stress disorder (F43.1)	1	4.2	1	4.0			
	Somatoform disorders (F45)	5	20.0	3	12.5			
Disorders of adult personality and behavior (F60-F69)	Emotionally unstable personality disorder (F60.30, F60.31)	2	4.1	4	7.5			
Substance abuse (F10, F12, F15, F19, F55)	Total	0	0.0	2	8.0			
Other mental disorders (F60.5, F60.8, F63.8)	Total	2	8.3	1	4.0			
Intake of anti-depressants	Pre-test	10	40.0	12	50.0	.48
	Post-test	11	44.0	6	25.0	.16
**Bio-psycho-social factors**								
Psycho-social factors	Occasional alcohol consumption	19	76.0	19	79.2	.90	
	Regular alcohol consumption	2	8.0	1	4.2	*Chi²-*test not performed		
	Oral contraceptives	2	8.0	5	20.8			
	Exposure to chemical toxic agents	3	12.0	5	20.8			
	Smoking	4	16.0	7	29.2	.27		
Reported diseases	Frequent headaches	11	44.0	7	29.2	.28		
	Hay fever	8	32.0	8	33.3	.92		
	Frequent colds and flues	4	16.0	3	12.5	*Chi²-*test not performed		
	Hindered nasal respiration	3	12.0	4	16.7			
	Non-insulin-dependent diabetes	1	4.0	2	8.3			

The level of significance was set to.05 for all results; ^*^age, duration of disease, and number of diagnoses was only assessed at pre-test, ^**^as tested with the t-test for independent samples, ^***^as tested with the chi²-test, this test was only performed if the sample size in all fields was ≥5, ^+^as tested with the Mann Whitney U-test, ^++^ adjustment disorders with depressive reaction were included in the group of depression, ^+++^ as classified in the ICD-10 Version: 2010, http://apps.who.int/classifications/icd10/browse/2010/e.

BDI, Beck Depression Inventory; RWT-FL, Regensburger verbal fluency test.

Exclusion criteria were diagnosed anosmia, chronic nasal diseases, neurodegenerative diseases, and metabolic diseases, as well as being currently affected with acute respiratory diseases like colds and influenza, although frequent respiratory diseases in their medical history were not an exclusion criterion. Furthermore, current psychological treatment, antidepressant medication, or the admission into psychotherapeutic treatment facilities during the course of training was not an exclusion criterion due to ethical reasons, but was taken into account for in the statistical data analysis.

The two groups (OT vs CT) did not differ significantly concerning the intake of psychotropic drugs at pre-test, *t*(100) = .36, *p* = .72. In the OT group, six participants took SSRI on a regular basis, three participants took SSNRI, four took tricyclic, and six participants tetracyclic antidepressants, while two participants took St. John’s wort and one person atypical neuroleptics. In the CT group, nine took SSRI and three participants took SSNRI on a regular basis, one took tricyclic and five subjects were subscribed to tetracyclic antidepressants, while two participants took St. John’s wort.

We observed a high number of dropouts: 53 participants (52%; OT: *n* = 28; CT: *n* = 25) canceled the training after less than 3 months (for a detailed analysis, see results section). We therefore decided to analyze the data in two ways. All 102 participants were included in an intention-to-treat-analysis. The data of the 49 participants who completed the training was investigated in a protocol analysis.

Of these remaining 49 participants, thereof 30 females, aged 18–69 years (*M* = 39.2, *SD* = 12.6), 25 participants conducted the OT and 24 conducted the placebo training in a parallel trial design. They neither differed in age (OT group: *M* = 40.4, *SD* = 12.4, range: 20–60; CT group: *M* = 38.0, *SD* = 12.9, range: 18–69) nor sex (OT group: 17 females; CT group: 13 females) at pre-test (for a detailed sample description see **Table 1**). The patients were diagnosed with major depression, dysthymia, recurrent depressive disorders, and adjustment disorders with depressive reaction. There were comorbidities with other mental disorders in both groups and comorbidity was equally distributed among the groups, *t*(47) = .41, *p* = .68, *d* = .12 (for a detailed sample description see **Table S1**). The two groups were comparable concerning the intake of psychotropic drugs at pre-test: In the OT group, two participants took SSRI on a regular basis, two participants took SSNRI, one person was subscribed to tricyclic and one person to tetracyclic antidepressants, three participants took St. John’s wort and one person atypical neuroleptics. In the CT group, five participants took SSRI, three took tetracyclic antidepressants and three participants took St. John’s wort on a regular basis. The mean depression score as measured with the Beck Depression Inventory (42) did not differ between the two groups (see **Table 1**) and indicated on average moderate symptoms of depression at pre-test (OT group: *M* = 27.3, *SD* = 10.8, range: 13–50; CT group: *M* = 23.5, *SD* = 9.2, range: 14–49). Accordingly, the patients reported a high load of psychological symptoms in the short form health survey (SF-36; OT group: *M* = −2.0, *SD* = 0.8; CT group: *M* = −2.0, *SD* = .7) but not a high load on physical restrictions (OT group: *M* = −0.3, *SD* = 1.2; CT group: *M* = −0.3, *SD* = 1.0) compared to the normative sample (43). The olfactory function as assessed with the olfactory threshold and identification test (Sniffin’ Sticks; Hummel et al., 1997) did not differ between the two groups at pre-test (threshold: OT group: *M* = 9.7, *SD* = 3.5, range: 1.25–16.0; CT group: *M* = 10.9, *SD* = 3.4, range: 2.25−15,75; identification: OT group: *M* = 12.8, *SD* = 1.6, range: 10–16; CT group: *M* = 12.1, *SD* = 2.3, range: 5–16). In comparison to the age matched normative sample (44), both groups scored in the range of a medium olfactory function +/− one standard deviation and can hence be considered “normal”. Looking at the individual values, there were seven patients who exhibited ceiling effects (values of 15 or 16 out of 16) in at least one of the olfactory test scores at pre-test. No cognitive impairments were observed in any of the patients as measured with the d2 attention and concentration test (45) and with the Regensburger verbal fluency test [RWT, (46)], as none of the participants scored more than two standard deviations below the normative sample. The two groups did neither differ in cognitive function in terms of verbal flexibility (OT group: *M* = 14.0, *SD* = 3.6, CT group: *M* = 12.4, *SD* = 3.3) nor in attention (OT group: *M* = 191.0, *SD* = 42.7, CT group: M = 191.1, SD = 44.6) in the pre-test (see **Table 1**).

All participants were supplied with a detailed information sheet, provided written informed consent and received a moderate financial compensation. The study was conducted according to the Declaration of Helsinki (47) and was approved by the Ethics Committee of the Medical Faculty Carl Gustav Carus at the Technical University Dresden (EK 48022015). This clinical trial was conducted following the guidelines of the Consolidated Standards of Reporting Trials (CONSORT) statement and checklist (48).

### Material

Depressive symptoms were assessed using the BDI-II (42, 49). In this questionnaire, the severity of depressive symptoms during the last 2 weeks is self-reported based on 21 items presented on a four point Likert scale, ranging from 0 to 3 per item. Different anchors are used for each item, while a value of 0 always indicates the absence of a specific depressive symptom and a value of 3 the highest symptom severity. A score of >13 and <20 points indicates a mild clinical depression, whereas moderate depression ranges between a score of 20 and 28 and severe depression between 29 and 63. The BDI-II is characterized by good reliability and yields a coefficient alpha of .92 for the outpatient population (*n* = 500) in the normative sample referred to in the manual (42). Additionally, the 36-item Short Form Health Survey [SF-36, (43)] was applied in the pre-test to measure health-related quality of life. The SF-36 is a cross-disease measuring instrument covering eight dimensions, which can be conceptually classified into the areas of “physical health” and “mental health”: physical functioning, physical pain, role limitations due to physical health, general health perceptions, vitality, social functioning, role limitations due to emotional problems, and emotional well-being. The internal consistency of the subscales yields a Cronbach’s alpha between *r* = .57 and *r* = .94 (43).

Olfactory function in terms of odor threshold and identification ability was measured with a validated and reliable forced choice paradigm using the Sniffin’ Sticks testing kit [Burghart GmbH, for a detailed description see (50)]. In deviation to the trial protocol, we decided to use the 16 sticks version (“Version A”) instead of the 32 sticks version when measuring odor identification, in order to use the other 16 sticks (“Version B”) at post-test and thereby avoid recognition effects. The presentation sequence of Version A and B was alternated periodically for pre- and post-tests. In addition to the assessment of objective olfactory function, we also asked our participants about their individual importance of olfaction with a questionnaire (51). This inventory exhibits a good internal reliability (Cronbach’s alpha = .77) and consists of three subscales: odor-associated unconscious emotions and memories (subscale “association”), use of the sense of smell in daily life (subscale “application”), and consequences on behavior (subscale “consequence”).

In order to control for potential confounders like accompanying diseases, we included a medical history questionnaire. Here we asked for different diseases (“Do or did you suffer from any of the following medical conditions?”), such as frequent colds, hay fever, impaired nasal respiration, frequent headaches, neurological diseases, and diabetes, as well as alcohol consumption, smoking status, medication, and exposure to chemical toxic agents.

In order to control for a potential cognitive impact on olfactory function (52) and affective disorders (39), two cognitive tests were performed with the participants: The revised d2 attention and concentration test (45) measures speed and accuracy to distinguish visual stimuli, whereas the RWT measures formal lexical verbal fluency and semantic-categorical verbal fluency (46). For both inventories, high reliability and concurrent validity have been reported from various populations: the d2 test shows an internal consistency between *r* = .80 and .95, whereas the RWT exhibits an interrater-reliability for all subtests of *r* = .99. For the statistical evaluation we focused on the concentration performance value “GZ − F” in the d2-test and the results of the “FL”-subtest of the RWT-test as a measure of verbal flexibility.

### Procedure

**Figure 1:**
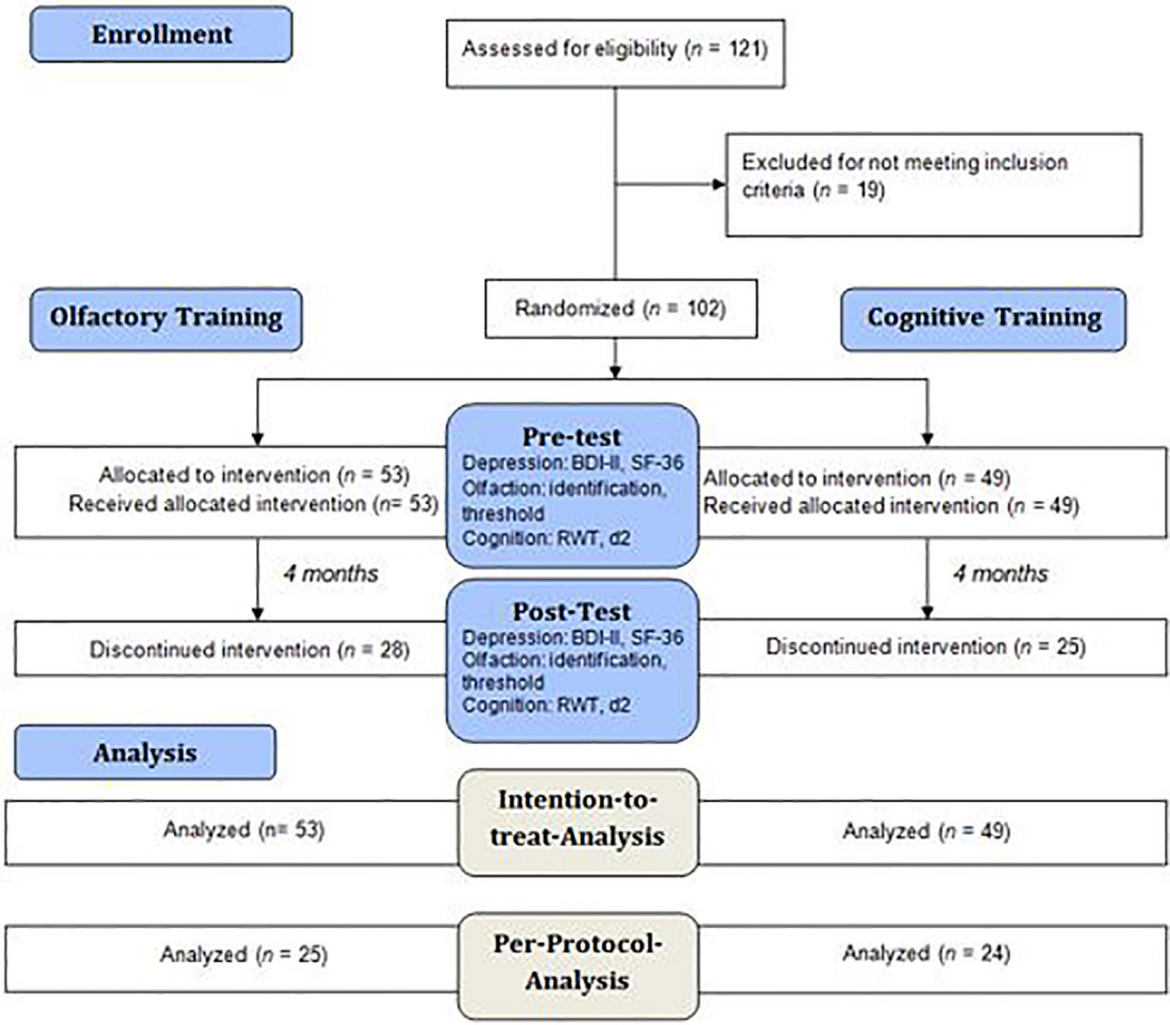
Flow diagram describing the analysis procedure in the experimental and the control group. All 102 participants were analyzed in an intention-to-treat analysis, however as *n* = 53 participants dropped out of the study, the remaining 49 participants were analyzed in a per-protocol analysis. The participants were tested for depressive symptoms, olfactory function and cognitive function at pre-test and post-test.

We ran a preliminary telephone interview to inquire the exclusion criteria. The study included two appointments consistently in the same quiet, well-lit, odorless consulting room with the same investigator in the psychosomatic outpatient department of the Clinic of Psychotherapy and Psychosomatics, University Hospital Dresden. Olfactory and cognitive function as well as subjective importance of olfaction and physical and emotional well-being were assessed using the same standardized tests both in the pre-test and in the post-test (see **Figure 1**). The experimental group conducted a 16-weeks OT two times daily for 5 min each, while the control group solved Sudokus in the same period of time. Both groups were provided with a test-kit, consisting of a training diary and, depending on the training group affiliation, either four bottled odors or a book of Sudoku which they were asked to take home in order to perform the training in their domestic environment. The participants of both groups were told that the purpose of our research project is to examine a potentially positive effect of the respective training on depressive symptoms and quality of life to increase compliance and to achieve the blinding of the participants of the control group.

We encouraged the participants to implement a daily routine in conducting the training and recommended a fixed time in the morning and in the evening. After 2 months, the participants were contacted by phone to check on their compliance and encourage them to keep up with the training.

The participants of the OT group smelled on four bottled odors of 3,5 ml lime, cloves, eucalyptus, and rose (citronellal, eugenol, eucalyptus, and phenyl ethyl alcohol; all odors from Sigma, Deisenhofen, Germany; for a detailed description see (25). In order to enhance the compliance, we chose only pleasant odors for the test kit. The participants were asked to note odor intensities, subjective efficiency, and mood over a week of training in a “smell diary” (24). The odors were rated as well perceivable during the whole training (see **Figure S2**). The CT group solved Sudokus (Pit Fox, “500 Sudoku zum Einstieg. Leicht bis medium”, udv, 2015, Germany). This condition was chosen as it was already successfully implemented in one of our previous studies (38). Based on our knowledge, there is no direct impact of solving Sudokus on a regular basis on the impact of depressive symptoms, however, there might be indirect effects: There are hints that Sudoku training can improve cognitive function (53, 54), while cognitive training was shown to reduce symptoms of depression (55, 56). This was considered a “nonspecific improvement effect” by the authors (55).

**Figure 2:**
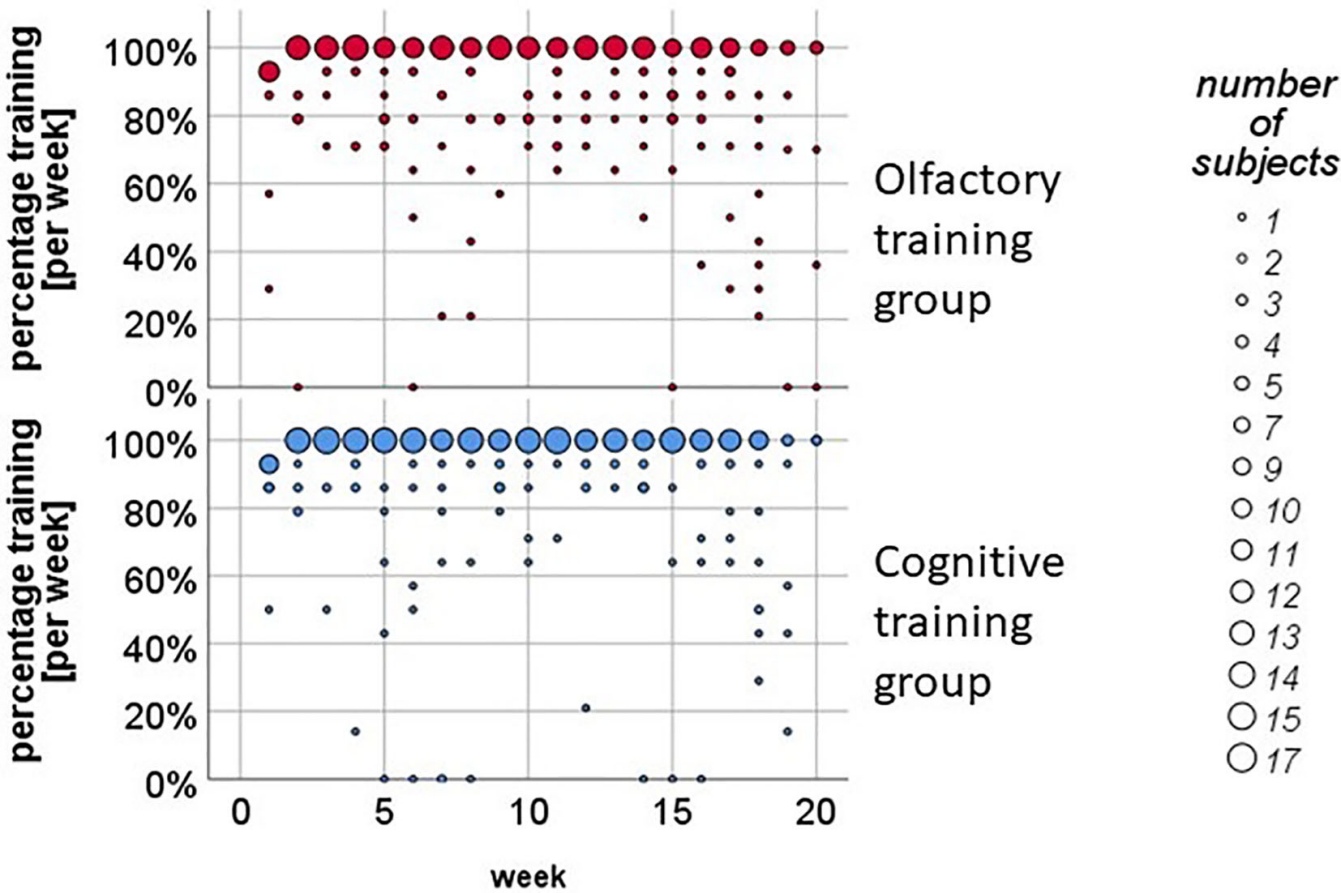
Weekly training analysis in the olfactory training (OT) and in the cognitive training (CT) group. About 70% of the participants pursued the training twice a day on a regular basis.

In the literature, the training period for OTs varies from 12 weeks (25, 26) up to 18 weeks and more (24, 57), so we decided to include all participants in the statistical data analysis that pursued the training continuously for at least 3 months. The 49 participants of the protocol sample pursued the training on average *M* = 3.94 months (*SD* = .20). The diary protocol revealed that about 70% of the participants completed the training for least 93% of the whole time-period (see **Figure 2**).

### Statistical Analysis

The statistical analysis was performed with SPSS (Version 25; SPSS Inc., Chicago, IL, USA). All data was investigated for normal distribution. The level of significance was set to 0.05 for all results and effect sizes are reported as η^2^ or Cohen’s d, respectively.

First, a dropout analysis was performed by comparing the pretest results in the group of patients who did not complete the training for at least 12 weeks to the group of patients who completed the training. The groups were compared according to age, depression score (BDI-II) and olfactory threshold and identification using a *t*-test for independent samples. The number of diagnoses and subjective olfactory function was compared using a Mann-Whitney *U* test. A *chi²-*test was used to compare the groups according to sex, intake of antidepressant medication, diagnoses (depressive disorders as well as comorbid neurotic, stress-related and somatoform disorders, disorders of adult personality and behavior, substance abuse, and other mental disorders), psycho-social risk factors (smoking, alcohol consumption, oral contraceptives, exposure to chemical toxic agents), reported diseases (frequent headaches, hay fever, frequent colds, hindered nasal respiration, non-insulin dependent diabetes), and current psychotherapeutic treatment.

Second, an intention-to-treat analysis, using the Last-Observation-Carried-Forward method (LOCF) was performed. In 14 participants who dropped out, post-test values were available. For the others, we conventionally assumed that there is no difference to the pre-test results (58). The effect of OT on depressive symptoms (BDI score) was investigated by a repeated measurement ANOVA with the within subject factor of time (pre-test vs post-test) and the between subject factor of group (OT vs CT). The main effect of time and the time by group interaction effect were modeled. In the next step, we analyzed the effect of OT on olfactory function. We therefore repeated the ANOVA and used olfactory threshold or odor identification, respectively, as dependent variable. Thereafter, the effect of OT on cognitive function was investigated in the same way and verbal flexibility or attention, respectively, were used as dependent variable.

Third, a per-protocol analysis was examined. Therefore, all steps which were performed in the intention-to-treat analysis were repeated with those 49 participants who completed the training conditions. We furthermore explored potential confounding effects. We therefore repeated the ANOVA with depression as dependent variable under A) exclusion of patients who were prescribed with anti-depressants, B) exclusion of patients who smoked, C) under exclusion of patients who suffered from recurrent depression, D) including concurrent psychotherapeutic treatment during the course of training as covariate, E) including the presence of comorbidity as covariate, F) including age as covariate, and G) splitting the sample for gender. Finally, we measured the relationship between olfactory function and depression score with a Pearson correlation and compared the eight participants that showed a significant improvement in odor threshold of at least 2.5 points (25) using *t*-tests for independent samples and *chi²-*tests.

## Results

### Dropout Analysis

The 53 participants (52%), who decided to drop out, did this mainly within the first month (compare **Figure S1**). We did not systematically access dropout reasons, but spontaneous reports of the patients indicated that a lack of motivation and private or professional stress load were major reasons for the participants to cancel their training. Two participants reported to drop out of the OT due to unintended side-effects during exposure to the odors. The reported side-effects comprised headache, dry mucous membranes, and a burning sensation in the nose, so that the concerning participants were encouraged to interrupt the training and, if required, make an appointment for a consultation in the smell and taste clinic of the department of ORL in our hospital.

The dropouts were equally distributed between training groups and did not differ from the remaining participants in age (*p* = .23), sex (*p* = .61), depression severity (*p* = .43), olfactory function (threshold: *p* = .70; identification: *p* = .18), number of diagnoses (*p* = .07), or intake of antidepressive medication (*p* = .59). However, dropouts had a lower number of ambulant treatment utilization (*p* = .02; for a detailed dropout analysis see **Table S1** in the **Supplementary Results**).

### Intention-to-Treat Analysis

Depression severity reduced over time in both groups by approximately 4 points, F(1, 100) = 30.00, *p* < .001, *η^2^* =.23, but no specific effect of training group (interaction effect) was observed: *F*(1, 100) =.01, *p* =.91, *η^2^* <.001. Olfactory threshold did not change over time, *F*(1, 100) =.06, *p* =.81, *η^2^* =.001, and there was no time by group interaction effect, *F*(1, 100) = 1.1, *p* =.34, *η^2^* =.003. Olfactory identification dropped slightly over time, *F*(1, 100) = 5.1, *p* =.03, *η^2^* =.05, and—again—we observed no significant time by group interaction effect: *F*(1, 100) =.10, *p* =.70, *η^2^* = 0.001). Verbal flexibility did not change over time, *F*(1, 100) =.08, *p* =.78, *η^2^* =.001, and there was no significant time by group interaction effect, *F*(1, 100) =.20, *p* =.66, *η^2^* =.002. Attention improved over time, *F*(1, 100) = 47.22, *p* <.001, *η^2^* =.32, and—again—no time by group interaction effect was observed, *F*(1, 100) =.02, *p* =.90, *η^2^* <.001.

### Per-Protocol Analysis

**Figure 3:**
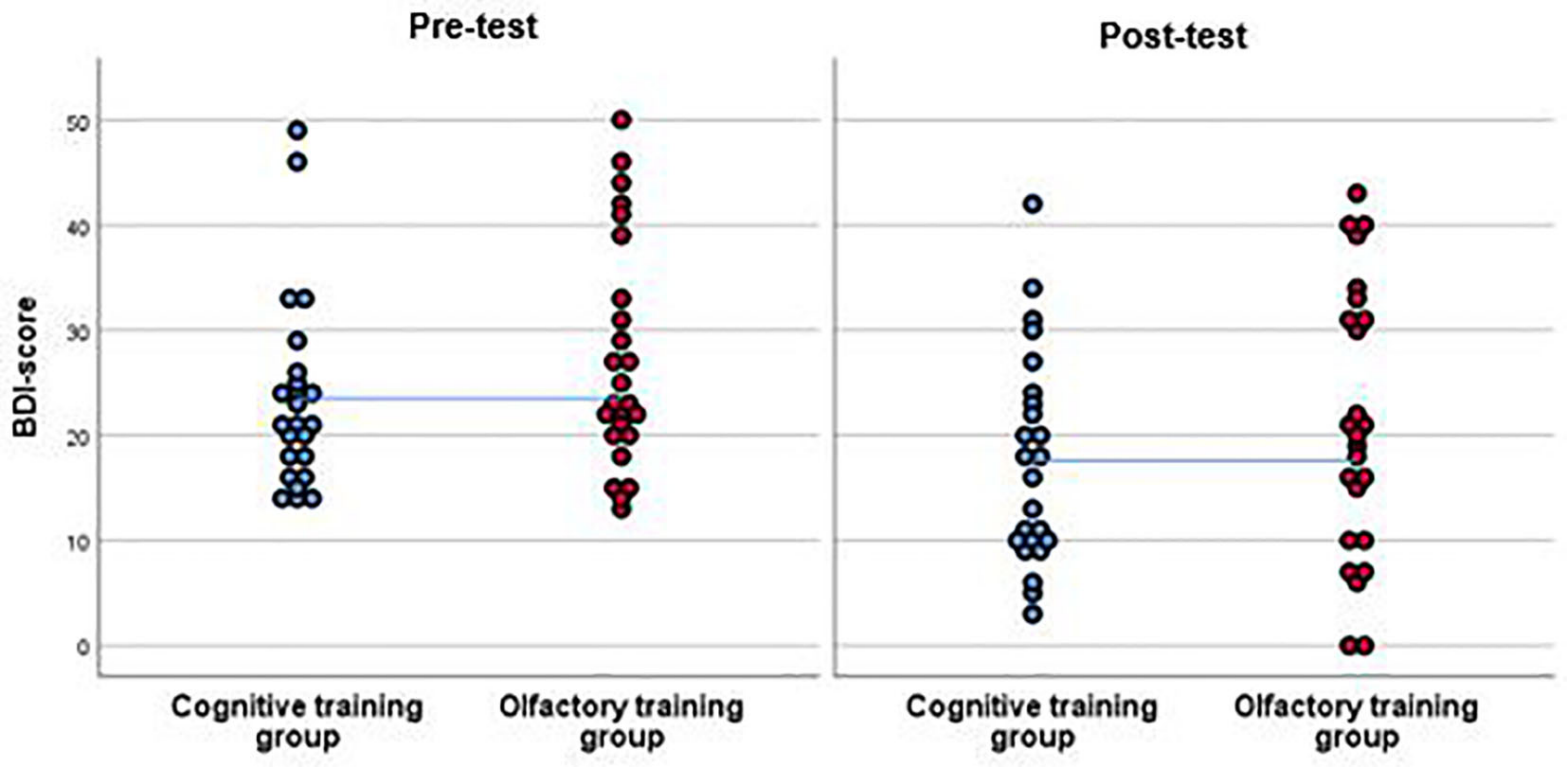
BDI-scores of the participants of the cognitive training group (CT) and the olfactory training group (OT) at pre-test and at post-test. Results from the per-protocol analysis are shown including 25 participants in the OT group and 24 in the CT group. Both groups improved in the post-test in terms of a reduction of BDI-score. This effect was significant. However, as the interpolation line shows, there were no significant differences between groups.

Depression severity decreased by of approximately 6 points among the patients, who completed either training, *F*(1, 47) = 24.7, *p* <.001, *η^2^* =.35, but no specific effect of training group was observed, interaction effect: *F*(1, 47) =.01, *p* =.93, *η^2^* <.001, **Figure 3**). In the attempt to not miss any potential effect, we repeated the ANOVA with different analysis strategies. None of those analyses revealed any significant group by time interaction effect. Hence, no such effect was observed when excluding A) patients who were taking anti-depressant medication, *F*(1, 25) =.04, *p* =.85, *η^2^* =.002, or who were B) smoking, *F*(1, 36) = 3.34, *p* =.08, *η^2^* =.09, a combination of those factors, *F*(1, 16) = 1.78, *p* =.20, *η^2^* =.10 or when C) excluding patients with recurrent depression, *F*(1, 20) =.25, *p* =.63, *η^2^* =.01. No significant interaction was observed when including the covariate of D) concurrent psychotherapeutic treatment, *F*(1, 46) =.09, *p* =.78, *η^2^* =.002, E) including comorbidity as a covariate: *F*(1, 46) =.04, *p* =.84, *η^2^* <.001, F) age, *F*(1, 46) =.01, *p* =.92, *η^2^* <.001, or G) looking at differential effects of gender; females: *F*(1, 28) =.01, *p* =.93, *η^2^* <.001; males: *F*(1, 17) =.01, *p* =.94, *η^2^* <.

**Figure 4:**
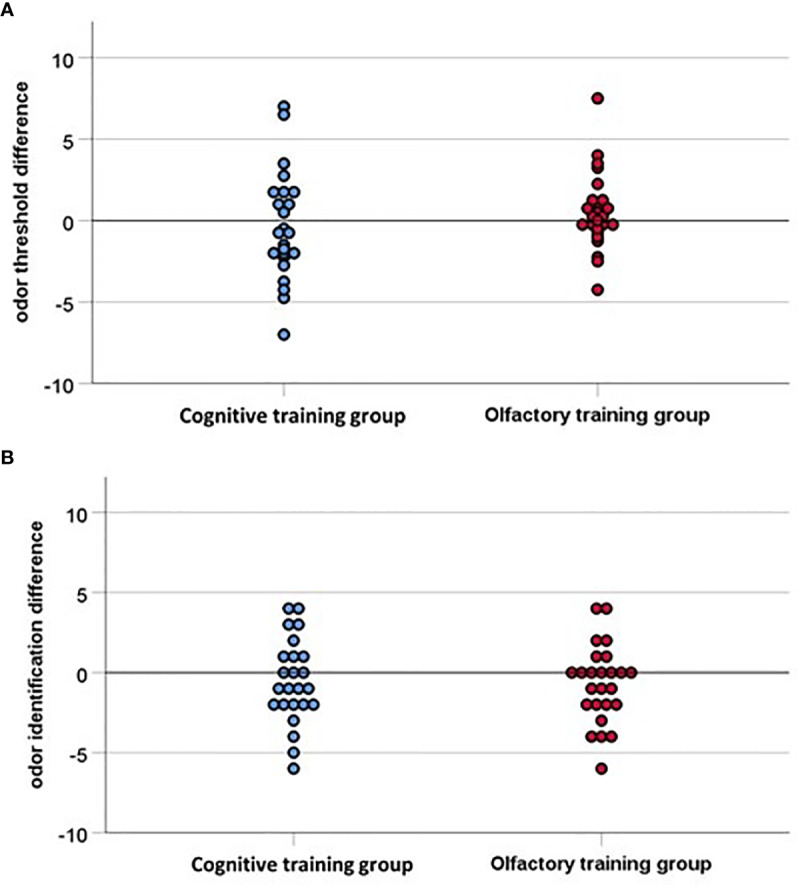
**(A)** Odor threshold difference and **(B)** Odor identification difference indicating the difference between pre-test and post-test (16 weeks later), with values above zero indicating an improvement in olfactory function. The interpolation line is close to the baseline for both groups, accordingly none of the groups improved significantly in the post-test, hence the two groups do not differ significantly.

Olfactory function did not change significantly over time, threshold: *F*(1, 47) =.04, *p* =.85, *η^2^* =.001; identification: *F*(1, 47) = 3.04, *p* =.09, *η^2^* =.06, and there were no significant time by group interaction effects: threshold: *F*(1, 47) = 1.10, *p* =.30, *η^2^* =.02; identification: *F*(1, 47) =.06, *p* =.80, *η^2^* =.001 (see **Figure 4**). Verbal flexibility did not change over time, *F*(1, 47) =.10, *p* =.87, *η^2^* =.001, and there was no significant time by group interaction effect, *F*(1, 47) = 1.70, *p* =.52, *η^2^* =.009. Attention improved over time, *F*(1, 46) = 51.50, *p* <.001, *η^2^* =.53, but again no time by group interaction effect was observed, *F*(1, 46) =.24, *p* =.62, *η^2^* =.005.

### Relationship Between Olfactory Function and Depression Score

We found a significant correlation (*r* =.28, *p* =.05) between odor threshold difference and BDI difference, both in the OT group and in the CT group, but only four participants in each group showed a significant improvement in odor threshold of 2.5 points and more. These eight participants did not differ in age (*p* =.27) or depression score (BDI pre-test: *p* =.60, BDI post-test: *p* =.70, BDI difference: *p* =.20. However they did differ in sex, *chi^2^* = 5.30, *p* =.02, odor threshold score at pre-test, *t*(47) = 3.10, *p* =.003, *d* = 1.20, and odor identification score at post-test, *t*(47) = 2.30, *p* =.02, *d* =.90, with more male participants, as well as participants with a lower odor threshold score at pre-test and participants with a higher odor identification score at post-test showing an improvement in odor threshold.

## Discussion

The present study examined the effect of OT on depressive symptoms and on olfactory function in a clinical sample. Although the diagnosis-inherent lack of motivation makes a high dropout likely, we were surprised to find that about half of the patients did not complete the training. For OT, the dropout rate is usually reported as significantly lower, for example five dropouts in a sample of 119 patients (57). As the dropout rate was equally distributed in both training groups, we assume that the training content did not contribute in great extent to the decision to drop out. The compliance to the training was facilitated by a low number in mental disorder comorbidity and lower psychotherapy utilization. Future research in this field could benefit from a rating of motivation added to the measures in the study design in order to closer examine a potential association between the index of motivation and dropout rate.

For those patients who completed the training, both the experimental group and the control group showed a reduction in depressive symptoms after an at least 12-weeks training period of approximately 23.6%. As some untreated episodes of major depression resolve after 3–4 months (59), this reduction of depressive symptoms in our sample is probably explainable by the effect of spontaneous remission. Specific effects of OT were not found in the intention-to-treat analysis. Also in the group of patients who completed the training, there was no a specific effect of OT on depression. The variance of depression improvement explained by the OT was as little as 0.1%, so even with a much larger group of depressive outpatients, we would not expect a significant effect of OT on depression. The effect we found was, with an effect size of η²<0.001, very small. In order to statistically ensure such an effect, over 2000 patients need to be examined [G*Power Version 3.1.9.2, (60)] and such effect is clinically not meaningful. Based on our study protocol, we cannot claim that OT is not useful, but it seems not more useful than the non-olfactory control condition.

We deliberately chose Sudoku as CT [compare previous studies (38)] to ensure a similar amount of frequency and duration of training in both groups. However, the training period may have provoked an unspecific effect of attention or activity which affected both groups and translated to a decrease of BDI score. Moreover, some researchers consider solving Sudokus on a regular basis as “cognitively stimulating leisure activity” and therefore as a form of successful cognitive training (53, 54). Also, cognitive training can have a positive impact on depression in terms of symptom reduction (55, 56). However, in our study design, we assumed no specific effect of Sudoku training on the reduction of depressive symptoms, which can be critically scrutinized.

Furthermore, there was no specific effect of OT on olfactory or cognitive function. Based on the previous study conducted in older patients (38), who exhibited olfactory dysfunction on average, we assumed that OT does not only improve olfactory function but also generalizes to an improvement of mood in patients with depression. We did intentionally recruit among outpatients in order to minimize the confounding effect of high intensity antidepressive treatment in inpatients. However, we did not observe a major olfactory impairment at the pre-test in the outpatients. In contrast and to our surprise, the baseline olfactory function assessment was already in the range of healthy controls (44). Patients with depression typically present reduced olfactory threshold and identification, however, most studies have examined depressive inpatients (10, 11). For outpatients, the effect seems to be less pronounced. We explain this finding with the heterogeneity of duration, course, and severity of depressive symptomatology in our sample (40). The high olfactory function at baseline may have caused a roof-effect, making a potential improvement of olfactory function more difficult to measure. It is unclear whether olfactory function can be improved in normosmic participants: Although there is evidence that repeated exposure to odors in healthy subjects significantly increases olfactory sensitivity (61–63), other research groups did not find an increase of olfactory sensitivity in healthy subjects (64, 65), or even a deterioration of odor threshold in normosmics after OT (66). As the mechanisms behind this phenomenon are still unclear, further studies are necessary to elucidate this contrasting finding.

As the olfactory function did not improve in our sample of depressive outpatients in the course of OT, a generalization to mood could not be expected. Another potential explanation for the lack of selective training effects is the reduced motivation in patients with depression which may have biased the performance of the training. This is reflected in the high number of dropouts. However, for the remaining participants, the analysis of the diaries showed a complying behavior. Alternatively, it can be suspected that depression severity among the patients in our sample was not pronounced enough to reveal an effect of OT which is stronger than the spontaneous remission. Therefore, especially severely depressed inpatients, which also show olfactory impairment, should be investigated before the idea of olfactory interventions for depression is abandoned.

Another option could be to incorporate OT as an additional “therapy tool” into the already existing psychotherapeutic treatment options for depressed patients, like cognitive behavioral psychotherapy, in order to monitor and ease the training period with the constant support of the attending psychotherapist, which could possibly improve the training outcome.

We also have to consider that OT, or olfactory interventions, are not suitable to improve depression at all. Although olfactory structures and those affected in depression show a high overlap and improvement of function in one area does not necessarily imply an improvement in the other one. Again, we feel that more research is needed in this area.

Our study is limited by the heterogeneous sample. Although this enhances the ecological validity of our study, it results in a large variance of severity, course, and duration of depression. Also, this sample of depressive outpatients could potentially underlie a self-selection bias. Furthermore, the unexpectedly high dropout rate due to lack of compliance dramatically reduced the sample size for the per-protocol analysis and disabled very specialized subgroup analyses. The higher rate of female participants compared to male participants in our study was recognized, but did not differ between groups, furthermore this distribution does not differ from the prevalence of depressive disorders in the population (67).

Based on our experience, for further studies examining a potential effect of OT on depression, we suggest: 1. Checking the compliance of our participants more frequently and encouraging them to continue the training more often to reduce the dropout rate. 2. Examining patients with a higher symptom severity of depression (inpatients and/or longer duration of depression), which makes it even more important to monitor potential dropouts closely. 3. Examining olfactory function beforehand and focus on those patients with hyposmia (against a control group with hyposmia). 4. Check for alternatives for the control condition: We learned that Sudoku is no ideal control condition, but we did not come up with a better alternative, as a strict placebo, like for example empty odor bottles, is very frustrating and likely reduces compliance. The advantage of Sudoku is the simple handling and its comparability with OT in terms of training interval duration.

### Summary and Concluding Discussion

Our findings indicate no beneficial effect of OT on depressive symptoms and on olfactory function for outpatients with affective disorders. Further studies may investigate different methods of olfactory stimulation, which are less dependent on an individual motivation.

## Data Availability Statement

The datasets generated for this study are available on request to the corresponding author.

## Ethics Statement

The studies involving human participants were reviewed and approved by Ethics Committee of the Medical Faculty Carl Gustav Carus at the Technical University Dresden (EK 48022015). The patients/participants provided their written informed consent to participate in this study.

## Author Contributions

LP, IC, and JM made substantial contributions to the conception and design of the present study. LP was responsible for the acquisition of data, whereas LP and IC contributed substantially to the analysis and interpretation of data. LP took the lead in writing the manuscript, while IC was supervising the project. IC, JM, KW, and TH revised the manuscript critically for important intellectual content. All authors contributed to the article and approved the submitted version.

## Funding

This work was financially supported by a MedDrive research funding granted to IC (MedDrive grant number 60.374).

## Conflict of Interest

The authors declare that the research was conducted in the absence of any commercial or financial relationships that could be construed as a potential conflict of interest.
