# Supplementary material for: Null Effect of Olfactory Training With Patients Suffering From Depressive Disorders—An Exploratory Randomized Controlled Clinical Trial
Source: Front Psychiatry. 2020 Jun 23;11:593. doi: 10.3389/fpsyt.2020.00593 (PMC7326271; doi:10.3389/fpsyt.2020.00593)
Supplement: Supplementary file 1 [file DataSheet_1.doc]

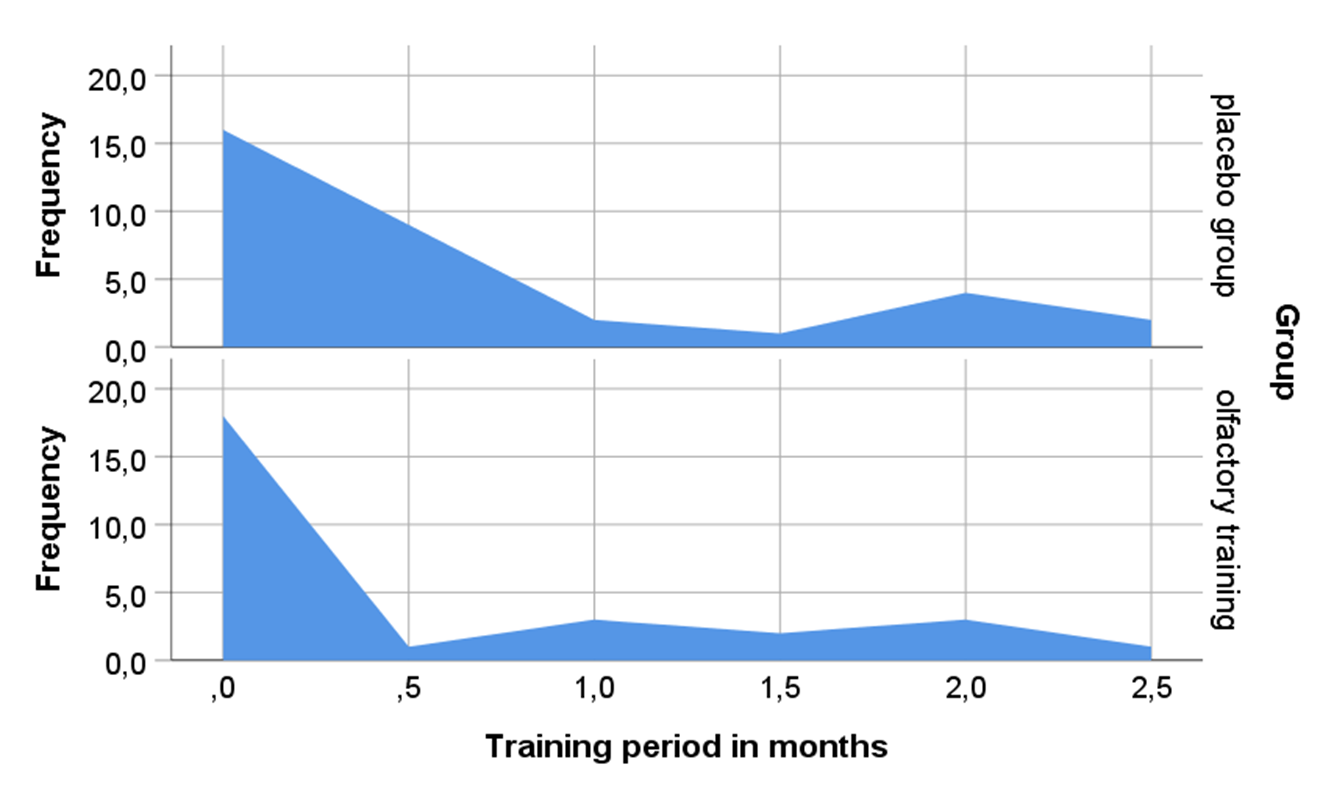


Fig S1. The participants that cancelled their training dropped out of the study mostly in the first month of training, with OT dropouts showing a higher dropout within the first two weeks compared to the CT dropouts.
