# Supplementary material for: Null Effect of Olfactory Training With Patients Suffering From Depressive Disorders—An Exploratory Randomized Controlled Clinical Trial
Source: Front Psychiatry. 2020 Jun 23;11:593. doi: 10.3389/fpsyt.2020.00593 (PMC7326271; doi:10.3389/fpsyt.2020.00593)
Supplement: Supplementary file 2 [file DataSheet_2.doc]

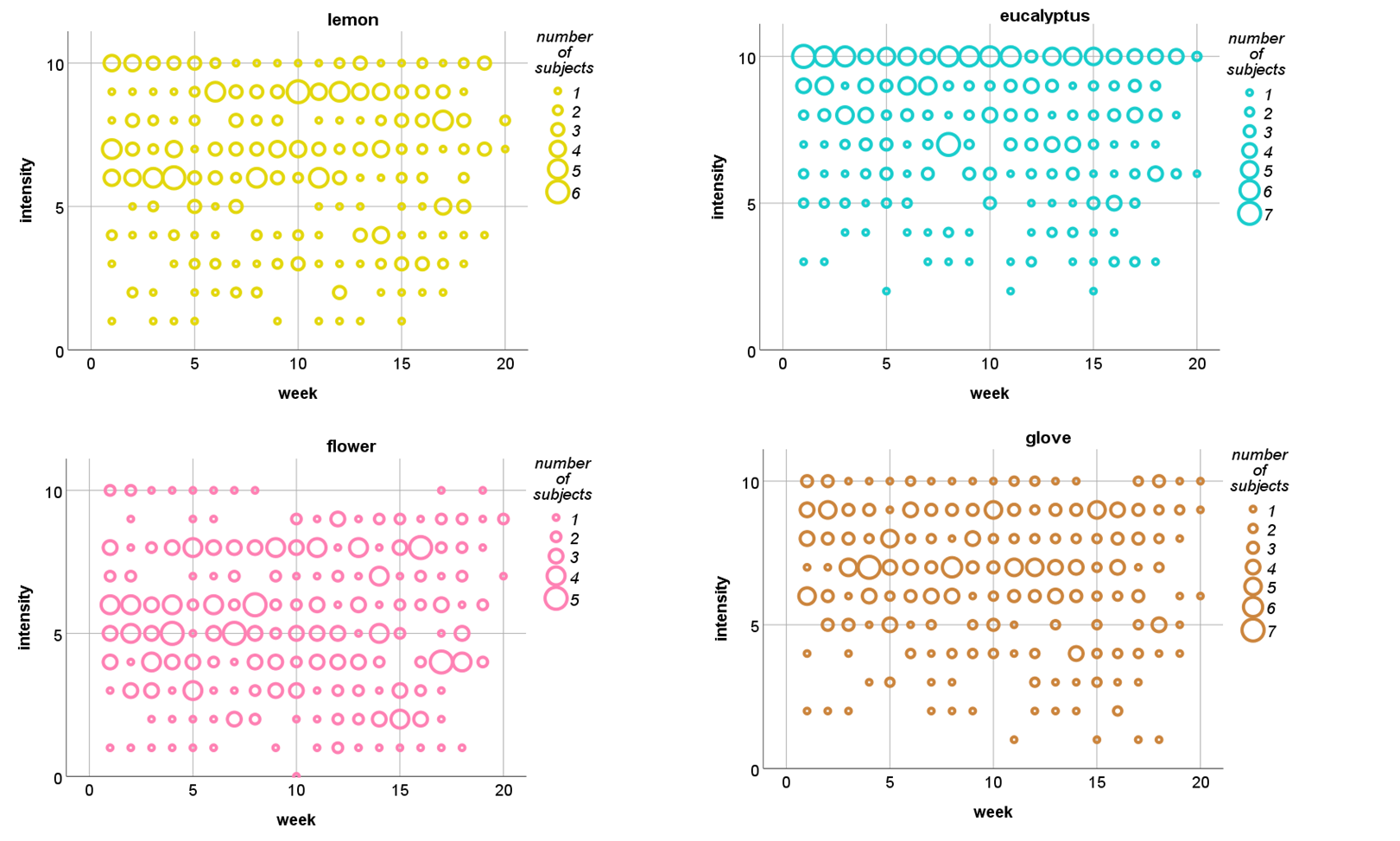


Fig S2. Intensity ratings within the group of patients performing the olfactory training of the four bottled odors lemon, flower, eucalyptus and clove.
