# Supplementary material for: Null Effect of Olfactory Training With Patients Suffering From Depressive Disorders—An Exploratory Randomized Controlled Clinical Trial
Source: Front Psychiatry. 2020 Jun 23;11:593. doi: 10.3389/fpsyt.2020.00593 (PMC7326271; doi:10.3389/fpsyt.2020.00593)
Supplement: Supplementary file 3 [file Table_1.doc]

Table S1. Sample description of the dropouts and comparison to the participants that finished the training period of at least 12 weeks, assessed at pre-test.

|  |  | **Participants (n = 49)** | | **Dropouts**  **(n = 53)** | |  | |
| --- | --- | --- | --- | --- | --- | --- | --- |
|  |  | ***M*** | ***SD*** | ***M*** | ***SD*** | ***p^*^*** | ***d*** |
| Depression severity (BDI) |  | 25.4 | 10.1 | 27.0 | 9.3 | .43 | .17 |
| Odor threshold |  | 10.3 | 3.5 | 10.0 | 3.2 | .70 | .09 |
| Odor identification |  | 12.4 | 1.9 | 11.9 | 1.9 | .18 | .27 |
| Importance of olfaction |  | 1.7 | 0.4 | 1.8 | 0.4 | .43 | .25 |
| Verbal fluency (RWT-FL) |  | 13.2 | 3.6 | 11.4 | 3.9 | **.02** | **.48** |
| Attention (d2) |  | 191.1 | 43.2 | 177.6 | 44.9 | .13 | .31 |
| Subjective olfactory function |  | 3.8 | 0.8 | 3.7 | 1.0 | .72~ | .11 |
| Age |  | 39.2 | 12.6 | 36.2 | 12.6 | .23 | .24 |
| Duration of disease in months |  | 37.5 | 51.5 | 51.4 | 104.3 | .40 | .17 |
| Number of diagnoses |  | 1.7 | 0.9 | 2.0 | 1.0 | **.07**~ | **.32** |
|  |  | ***n*** | ***%*** | ***n*** | ***%*** | ***p^+^*** |  |
| Sex (female) |  | 30 | 61.2 | 35 | 66.0 | .61 |  |
| Currently in psychotherapeutic treatment | | 13 | 26.5 | 5 | 9.4 | **.02** |  |
| **Diagnosis^+++^** |  |  |  |  |  |  |  |
| Mood disorders  (F30-F39) | Depressive episode (F32) | 15 | 30.6 | 22 | 41.5 | .25 |  |
|  | Recurrent depressive disorder (F33) | 22 | 44.9 | 27 | 50.9 | .54 |  |
|  | Adjustment disorders (F43.2)^++^ | 12 | 24.5 | 5 | 9.4 | **.04** |  |
| Neurotic, stress-related and somatoform disorders  (F40-F49) | Anxiety disorders (F40, F41) | 10 | 20.4 | 13 | 24.5 | .62 |  |
|  | Obsessive-compulsive disorder  (F42, F60.5) | 3 | 6.1 | 3 | 6.1 | *Chi²-*test  not per-  formed |  |
|  | Post-traumatic stress disorder (F43.1) | 2 | 4.1 | 5 | 9.4 |  |  |
|  | Somatoform disorders (F45) | 8 | 16.3 | 12 | 22.6 | .42 |  |
| Disorders of adult personality and behavior (F60-F69) | Emotionally unstable personality disorder (F60.30, F60.31) | 2 | 4.1 | 4 | 7.5 | *Chi²-*test not per-formed |  |
| Substance abuse (F10, F12, F15, F19, F55) | total | 2 | 4.1 | 8 | 15.1 | *Chi²-*test not per-formed |  |
| Other mental disorders (F60.5, F60.8, F63.8) | total | 3 | 6.1 | 4 | 7.5 |  |  |
| Intake of anti-depressants |  | 22 | 44.9 | 24 | 45.3 | .97 |  |
| **Bio-psycho-social factors** |  |  |  |  |  |  |  |
| Psycho-social factors | Occasional alcohol consumption | 38 | 77.6 | 31 | 58.5 | .14 |  |
|  | Regular alcohol consumption | 3 | 6.1 | 4 | 7.5 | *Chi²-*test not performed | |
|  | Oral contraceptives | 7 | 14.3 | 8 | 15.1 | .88 |  |
|  | Exposure to chemical toxic agents | 8 | 16.3 | 8 | 15.1 | .90 |  |
|  | Smoking | 11 | 22.4 | 21 | 39.6 | **.05** |  |
| Reported diseases | Frequent headaches | 18 | 36.7 | 30 | 56.6 | **.04** |  |
|  | Hay fever | 15 | 30.6 | 6 | 11.3 | **.02** |  |
|  | Frequent colds and flues | 7 | 14.3 | 11 | 20.8 | .37 |  |
|  | Hindered nasal respiration | 7 | 14.3 | 6 | 11.3 | .68 |  |
|  | Non-insulin-dependent diabetes | 3 | 6.1 | 1 | 1.9 | *Chi²-*test not performed | |

*Note*. The level of significance was set to .05 for all results; ^*^as tested with the *t*-test for independent samples, ~as tested with the Mann-Whitney *U*-test, ^+^as tested with the *Chi²-*test, ^++^ adjustment disorders with depressive reaction were included in the group of depression, ^+++^ as classified in the ICD-10 Version: 2010, http://apps.who.int/classifications/icd10/browse/2010/en.
